# From ‘support’ to ‘separate’: residential aged care staff responses to an intimate relationship involving a resident with cognitive impairment

**DOI:** 10.1186/s12877-025-05865-1

**Published:** 2025-04-04

**Authors:** Linda McAuliffe, Deirdre Fetherstonhaugh, Maggie Syme

**Affiliations:** 1 Australian Centre for Evidence Based Aged Care (ACEBAC), La Trobe University Melbourne Campus, Victoria, 3086 Australia; 2 Hebrew SeniorLife/Harvard Medical School, Marcus Institute for Aging, Roslindale, MA USA

**Keywords:** Sexuality, Intimacy, Cognitive impairment, Dementia, Nursing homes, Decision-making

## Abstract

**Background:**

Intimate relationships are important throughout life but can be complicated in later years if a person develops cognitive impairment and moves into residential aged care. The aim of this research was to elucidate the ways in which residential aged care staff would intervene in an intimate relationship between residents when a resident has cognitive impairment, and their motivations for intervening.

**Methods:**

This was an exploratory study. Vignette methodology was employed depicting a hypothetical case of a relationship between two residents (one who has cognitive impairment). A national postal survey was sent to all residential aged care facilities in Australia. Thematic analysis was performed on responses (*N* = 515) to open-ended questions regarding how staff would intervene and motivations for doing so.

**Results:**

Thematic analysis identified six key themes emerging from the data related to staff responses: communicating; educating; respecting; monitoring; distracting; and separating. Each of these themes is illustrated by participant quotes. Participants largely did not report motivations for responding in the ways identified.

**Conclusions:**

There is considerable variability in residential aged care staff responses to an intimate relationship between residents when one resident has cognitive impairment. Intimate relationships between residents can be supported or prevented as a result.

## Background

Sexuality and intimacy remain important to many people as they age, including older people living in residential aged care facilities (RACFs) [4]. This includes the estimated 50–87% of aged care residents who live with dementia [2, 21, 27]. Despite this, residents’ right to sexual freedom and expression is rarely supported in practice, with a ‘protective’ care paradigm often prevailing [16, 28]. Compounding the problem is a notable absence of policy to guide staff in many, if not most, RACFs [23, 25], and a lack of staff training, with many staff having never undertaken education in the area [25, 34].

Dementia can affect decision-making and consent capacity and there is often a lack of legal guidance to determine sexual consent capacity for older people who live with cognitive impairment [20, 32, 36]. RACF staff commonly assume that a diagnosis of dementia by default cancels the ability of the person to consent to, and engage in, intimate relationships and so adopt an ‘extreme cautionary stance’ when it comes to dementia and intimacy, possibly in an attempt to protect residents and manage perceived risk [37]. However, many people with dementia remain capable of making their own sexual decisions, and their sexual rights ought be upheld for as long as possible [15, 20]. Although dementia may complicate intimate relationships, it is still possible to tell the difference between “healthy and unhealthy, or wanted versus unwanted sexual behaviour” ([14], p. 27).

In the absence of formal policies regarding sexual expression and dementia in residential aged care, the personal views and values of staff have been found to guide practice (e.g., [26]). This is problematic, as there is considerable diversity in professionals’ attitudes regarding the sexual expression of people living with dementia [13]. This diversity stems from many factors, including personal sexual experience, religiosity, experience in aged care, and level of training in sexuality and dementia [17]. The impact of these attitudes is that they can play a pivotal role in either facilitating or constraining sexual expression in residential aged care [22].

Vignette methodology has previously been used to examine staff reactions towards partnered sexual expressions involving people with dementia living in residential aged care overseas [13, 38]). A study involving 2,295 staff members across 152 Spanish long-term care facilities found that staff viewed relationships involving persons with dementia as potentially problematic, requiring discussion with a colleague or supervisor [38]. Findings of a similar study involving 538 staff members across 28 Portuguese long-term care facilities echoed these results, with situations involving a resident with dementia perceived as difficult [13]. Interestingly, both studies found staff reactions to be more restrictive when only one person in the relationship had dementia.

The current study set out to discover whether staff reactions would be similar in the Australian context. Previously, we published that the likelihood of aged care staff in Australia indicating that they would intervene in a new intimate relationship between residents was heavily related to contextual factors, such as level of cognitive impairment, distress, and family involvement [26]. We also found support for the notion that personal views and the values of staff likely guide practice in the absence of formal policies in Australian residential aged care. What remains to be examined is what this value directed practice looks like.

This paper reports on the ways in which RACF staff indicated they would go about intervening in an intimate relationship between residents when one resident has a cognitive impairment, and the motivations they reported behind doing so. This paper addresses a research gap regarding how sexual relationships involving cognitive impairment are approached by staff in Australia.

## Methods

### Study design

Vignette methodology was adopted for this exploratory study. Vignette methodology is often used to examine decision-making processes by clinicians and can be highly generalisable to ‘real-life’ situations while overcoming the ethical and practical shortcomings of other methods, such as observation [12]. The vignette used in this study was constructed using research findings relating to perceived appropriateness of sexual/intimate behaviours in RACFs (see [35]) and was previously employed in a study evaluating future resident preferences [33].

The vignette (see Table 1) describes a hypothetical case of two older adults (a male with mild cognitive impairment who has a wife living in the community, and a female with no cognitive impairment) living in a RACF who develop a mutually satisfying intimate relationship consisting of mild physical contact (hand-holding, embracing, and kissing). Respondents were asked to reflect on whether they would intervene in the relationship as described.

**Table 1:** Vignette describing hypothetical case of an intimate relationship in aged care (from McAuliffe, Fetherstonhaugh, & Syme [26])

Norm moved into an aged care facility a year ago after his health deteriorated, and he required more care than the family could provide at home. Norm has some symptoms of dementia (mild cognitive impairment) as he forgets names of his usual care staff, has difficulty recalling his daily activities to his children and has started to unknowingly repeat the same stories to the same people. Norm and Carol are companions. Carol came to the facility around the same time as Norm. She was admitted there due to declining physical health and has no cognitive impairment. Since she came to the facility, Norm and Carol have developed a close relationship and they are rarely seen apart. Norm and Carol are observed to enjoy each other’s company, and Norm appears very happy when he is with Carol. He always asks for her to be by his side. Staff have observed that Norm and Carol spend much of their time together; Carol holds hands with Norm, cuddles him and occasionally gives him kisses.	

Open-ended questions invited those respondents who indicated they would intervene in the situation to describe what actions they would take and then to elaborate on the factors that influenced their decision to intervene *–* these responses are the focus of the current paper. Open-ended questions are useful in vignette studies and can be advantageous as they “allow participants to respond to the vignette in different ways” ([1], p. 2). Quantitative findings relating to a number of contextual variations of the vignette and associated intervention decisions have been reported elsewhere [26].

### Participants

Respondents (*N* = 515) were senior staff (e.g., facility managers (FM; 64%); nurse unit managers (NUMs; 14%); or other senior staff (22%)) working in public (government; 18%), private (for-profit, 27%), or not-for-profit (55%) RACFs in Australia. Most identified as female (88%) with a mean age of 51 years (*SD* = 10.80), reflecting the general profile of senior staff working in the area. Most were currently married or partnered (74%). On average, respondents had worked in aged care for 16 years (*SD* = 9.51). The majority of participants had received education on sex and the older adult (79%) and in the course of their work had addressed a situation relating to resident sexuality (89%). Just over half (55%) worked in a RACF with a written policy on sexuality.

### Data collection

The vignette and accompanying questions were posted to all 2931 RACFs in Australia. Envelopes were addressed to the facility manager or nurse unit manager as they represent more senior staff working in aged care and are therefore more likely to have encountered a situation involving resident sexuality. Data collection occurred over an eight-week period. The study was performed in line with the principles of the Declaration of Helsinki and was approved by the La Trobe University Human Research Ethics Committee (Application No. HEC18483) prior to commencement.

### Data analysis

Qualitative thematic analysis of staff responses to the open-ended questions was conducted using established methods [7, 8]. In the initial stages, a subsample of staff responses was independently manually coded by two members of the research team (LM and DF) for basic themes, with consensus reached before conducting further analysis [24]. The second stage involved two members of the research team reading and re-reading all participant responses (LM and DF), independently performing manual coding; examining codes and identifying broad themes; reviewing preliminary themes; final refinement and defining of themes; and writing up of themes [7, 8]. Participant quotes were extracted from the data to illustrate each theme.

## Results

Six key themes emerged from staff responses: communicating, educating, respecting, monitoring, distracting, and separating. These themes are elaborated upon below. Participant quotations illustrative of the themes are attributed to individual participants using unique identifiers (FM = Facility Manager, NUM = Nurse Unit Manager, OSS = Other Senior staff; f = female, m = male, n = non-binary; Xy = number of years worked in aged care).

### Communicating

Communicating was a main theme to emerge from the responses, with respondents indicating that they would intervene by communicating directly with one, or both, of the residents; with the residents’ families; with other aged care staff; or with other health professionals, usually the residents’ general practitioner (GP) or geriatrician, or less commonly, dementia specialist team, psychologist or sexual therapist/counsellor. Respondents indicated they would communicate in most cases with a combination of parties. Only some respondents stated that they would have a conversation with the residents themselves in the first instance.

*Family conference involving residents. GP involvement. Discussion with multidisciplinary team.* (FM297_f18y)

*Discuss with both residents and their NOKs [Next of Kin]. Make sure all staff are aware.* (NUM419_f15y)

*Firstly, communicate with the residents.* (FM229_f10y)

The reasons provided for intended communications varied depending on the parties involved. Communication with residents largely centred around ways to support the residents achieve their intimacy needs, but also included discussions about dementia and consent. In contrast, communication with staff and family mainly involved education on ageing/dementia and intimacy, explaining residents’ rights, and ensuring staff were working from a paradigm that respected resident choice. Communication with the GP and geriatrician was usually for the purpose of seeking advice regarding capacity and consent.

*We would talk to the residents about their feelings and what they want and implement the strategies to help them achieve their goals*. (FM17_f11y)

*I would contact the family to discuss [the] relationship and organise a meeting with family and our DON (FM) [Director of Nursing; Facility Manager]. This is to ensure that all parties involved are informed and understand residents’ rights and facility responsibilities to protect (advocate for) the residents*. (NUM172_m7y)

*Discussing with medical power of attorney/treatment decision maker on the relationship explaining everyone has a right to intimacy*. (NUM33_f27y)

Respondents generally relayed the intended content of communications only in broad terms. For example, while they mentioned they would ‘discuss’ with residents and others, and implement ‘strategies’, little detailed information was provided about what these discussions and strategies would encompass. An exception to this was a respondent who stated they would intervene by *“requesting the legal partner to visit regularly and encourage attending to resident’s sexual needs”*.

### Educating

Related to communicating, educating was a second main theme to emerge from responses. Educating was primarily mentioned in the context of families and staff, for the purpose of increasing their knowledge in the areas of ageing/dementia and sexuality (including associated potential benefits to mental health and quality of life), residents’ rights/choice, and consent. Education was viewed as a means of overcoming initial disapproval of, or resistance to, the residents’ relationship. Education for residents was also mentioned, but less frequently, and focused largely on consent.

*Family education around intimacy and aging, considering cognitive ability, social, emotional and cultural needs of both parties involved. Staff education on intimacy in ageing.* (NUM311_f13y)

*If family members are upset, would hold a discussion with them and provide education on sexuality and intimacy. Would advise them the relationship will be monitored and if signs of distress from one party, then action will be taken, otherwise no action will be taken.* (FM476_f15y)

*Education to other staff especially around consent and rights*. (NUM434_f14y)

*Educate resident on signs of consent withdrawal*. (FM251_f25y)

Interestingly, education about physical sexual health was largely missing from responses. Few respondents mentioned the provision of sexual health information (including safe sex practices) to residents as an intervention they would adopt.

*Sexual health education – hygiene?* (FM136_f26y)

*Engage a sexologist to discuss safe sex practices, provide education/information and include the family members*. (FM281_f10y)

### Respecting

Respecting was another strong theme to emerge from responses. In relation to the residents in the relationship, respondents spoke about respecting the residents’ privacy, dignity, and right to make decisions around intimacy *–* despite mild cognitive impairment. Respecting also involved staff being discrete when responding to the relationship in order to avoid ‘gossip’ and maintain resident confidentiality.

*A relationship between two people is all about how it makes them feel, whether they have cognitive impairment or not. If they are both happy, feel safe, then I would support their relationship and provide privacy as needed.* (FM354_f30y)

*Be respectful, tactful and supportive. Promote relationships and intimacy if consent is apparent. Support this to happen in a safe way. These situations are always very sensitive and should be treated with the utmost respect for all parties. Confidentiality is very important.* (FM250_f12y)

*Well-considered and discreet intervention, respecting dignity and choice, decision-making capacity.* (OSS423_f20y)

*Encourage friendship and participation.* (FM50_n15y)

Respecting extended to supporting the resident relationship. Some – but not many – respondents identified practical ways in which they could help optimise conditions for resident intimacy. These almost exclusively related to privacy. Only one respondent mentioned the provision of protection as an intervention.

*Would ensure privacy for the couple. Remind staff to respect closed doors, knock before entering a room.* (NUM195_f20y)

*Provide privacy* – *signs for doors, "Do not disturb".* (FM346_f40y)

*Provide education and required equipment. Intervene to provide education or to minimise harm only.* (OSS83_f1y)

The theme of respecting also arose in relation to the residents’ families, including the appointed medical treatment decision maker or power of attorney (POA). Some respondents reported that they would respect the wishes of the resident’s POA – even when this was in direct conflict with the current wishes of the resident. Another reflected on the complexity involved in decision-making when a resident has cognitive impairment.

*[Intervention] would also depend on family and their thoughts, perceptions etc*. (FM518_f5y)

*If their POA was making the decisions theirs would be respected on behalf of the resident.* (FM401_f22y)

*It’s very sad how NOKs dictate what is appropriate or not for their loved ones with dementia or not. Feel like EPOA* [Enduring Power of Attorney] *gives them the right to manage everything. I feel that public education is needed about elder rights including intimacy.* (FM65_f20y)

Respecting also extended to other residents and staff. Respondents cited the perceived impact of the relationship on other residents (and to a lesser extent, staff) as a reason they would intervene in the relationship. While some respondents explicitly stated that would be prompted by inappropriate activity, such as activity occurring in a communal living area, others indicated they would intervene in response to resident views/reactions more generally.

*If they are totally disinhibited and it is affecting others’ wellbeing [intervention] may include transfer to [a] more appropriate setting*. (FM99_f15y)

*Does the relationship take place openly to the distress of others or do other residents take pleasure in the happiness and comment positively*. (FM15_f16y)

*I would intervene if other residents complain*. (NUM11_f20y)

*Might limit the 'public' side of affection*. (FM444_f25y)

*Potential impact of behaviour on other residents and staff.* (FM157_f5y)

### Monitoring

Monitoring was considered a form of intervention by some respondents, a type of ‘waitful watching’ or ‘wait and see’ approach. Respondents spoke of the need to ‘watch closely’ to ensure the relationship stayed a happy one and was not causing either resident harm/distress. Monitoring was also considered necessary to ensure ‘inappropriate behaviour’ did not occur in common areas or that other residents were not impacted by the relationship.

*I would watch closely however while they are happy and not hurting anyone else why should it matter. They have rights, dignity and choice*. (FM427_f28y)

*I would only intervene if there was a risk of harm to either party, but even then initially increase supervision to establish their triggers.* (FM7_f21y)

*Would be aware and monitor for any actions that may be interpreted as a violation of rights or abuse*. (OSS164_m15y)

*Monitor both residents for distress. Risk assess situation, identify support for each resident. Discuss with family* – *outline expectations, support. Communicate actions, support and outcomes to staff*. (FM499_male10y)

### Distracting

Staff reported that they would intervene by diverting the residents’ attention away from the relationship. Few respondents elaborated on how this would be achieved; those who did so stated that they would offer alternative activities.

*Distraction* – *attempt to engage them in activities separately*. (FM10_f7y)

*Distraction* – *offer food, drink* – *other activity in facility.* (NUM457_f10y)

*I would work with lifestyle welfare to implement new strategies/activities*. (FM14_f18y)

Only one respondent provided a more detailed description of the form that possible distractions might take.

*Re-direction. Sensory distraction such as massage and aromatherapy. Intellectual stimulation, conversation and activity. Music and reminiscence.* (FM484_f30y)

Motivations for distracting residents from engaging in the relationship were not reported.

### Separating

Separating the residents was a step many respondents reported they would take, often as a first step intervention. The extent of the separation ranged from sitting one of the residents at a different dining table to moving one of the residents to another wing of the facility.

*Still married. Move residents to separate lounges and tables*. (FM297_f15y)

*Ensure that they are placed in different wings*. (OSS3_f7y).

*Divert. Keep separated as much as possible*. (OSS90_f3y).

Family was frequently mentioned in tandem with the expressed intent to separate residents. This could either be in terms of informing the family that residents had already been separated, or deferring to the family’s wishes when considering separation as an intervention.

*Ensure the residents are placed in different wings. Explain to residents and family. Seek advice from resident’s NOK/wife*. (OSS3_f7y)

*Separate, diversion and engagement of activities for both residents (if family wish etc)* (FM103_f25y)

*Attempt to separate residents. Definitely talk to family first try to work things out with them. Difficult situation but if the person was married it is important to maintain family stability by ensuring the situation is managed by stopping behaviour.* (NUM447_f30y)

Several respondents specifically commented that the residents should not be separated as they appear to be content as described in the vignette. The further point was made that such intervention could have the unintended consequence of inducing resident distress.

*Ultimately if the individuals are happy with the relationship, no intervention should be made to separate them. If the family are not happy with this relationship, discussions should be made with them, and if needed, counselling to be provided/arranged.* (OSS473_f6y)

*If the residents are both happy and consenting then who are we to step in and ultimately cause distress?* (FM42_f12y)

*Not to intervene* – *they are consenting adults and are enjoying each other’s company. Neither are displaying any distress/agitation. The distress and agitation would develop if they were separated. They are not hurting anyone. Everyone has a right to express themselves in this way.* (NUM79_f25y)

## Discussion

As highlighted by Roelofs and colleagues, “although challenging to appropriately facilitate at RCFs (residential care facilities), love, intimacy and sexuality are still important aspects for residents with dementia…” ([29], p. 288). This study set out to identify how staff would respond to an intimate relationship between residents when a resident has cognitive impairment, and their reasons for doing so. The findings of this study are that aged care staff report a variety of ways in which they would intervene in a resident intimate relationship involving cognitive impairment, and that the nature of these intended interventions spans from supportive to prohibitive. While some staff prioritised the needs and rights of the residents, for many others distraction and separation were commonly cited first step measures, and the views of family featured heavily as a driving motivation for staff actions.

Decision-making is complex when a resident has cognitive impairment and there are questions around capacity and consent; these findings suggest that when faced with such complex decision-making, staff turn to others and adopt a team approach. This supports findings from a large Spanish study of long-term care facility staff which found discussing the case with a colleague or supervisor was the most frequently chosen reaction to sexual situations involving residents with dementia, possibly as a way of discharging responsibility [38]. Good communication has been found to have a positive impact on clinical outcomes among older adults [30], and this may also extend to resident sexual expression. Communicating with multiple members of the care team and with family who have raised concerns can help clarify expectations and understandings and ensure that everyone is ‘on the same page’ when it comes to supporting resident intimacy. Communicating also creates an opportunity for educating, which was another main theme to emerge from the findings. Knowledge around sexuality and ageing can be poor among families and even health professionals [17], and educating can help correct misperceptions, highlight potential health benefits, emphasise residents’ right to choose, and clarify issues of capacity and consent.

Also highlighted by these findings is that communicating with, and educating, residents was not common. When staff did indicate that they would talk to or educate residents, it was largely in the context of discussing consent. While discussions around consent are no doubt important and are needed in order to support safe sexual expression, this exclusive focus represents a real missed opportunity. Older people are typically not given any information on how their right to intimacy will be respected in aged care [6] and although many want to be invited to discuss their sexual needs with health professionals, this rarely happens in practice [5]. Additionally, older people are a cohort with an increasing prevalence of sexually transmitted infections (STIs), in part due to variable sex literacy, and need education around safe sex practices [9]. Initiating conversations with residents about how they can be supported in their new or existing relationship should be a first step.

Staff were more likely to indicate that they would communicate with families (rather than the residents) about the intimate relationship. While families may hold the roles of next of kin or medical power of attorney, and may be entrusted with medical decision-making, this does not extend to decisions of a sexual nature. There is no legal proxy for sexual consent [18], which brings into question why staff would feel the need to inform families. Previously, sharing information with relatives has been found to be an uncommon reaction to resident sexuality involving cognitive impairment [38], possibly due to fears of potential litigation, or not wanting to violate residents’ right to confidentiality [36]. It is unclear from our findings whether staff went out of their way to ‘report’ sexual activity to families or whether communication with family was prompted by the family member raising a concern. The findings do however suggest that staff motivation for communication with family may be driven by a perceived need to educate family about dementia and intimacy and residents’ rights. This represents an important shift towards a more resident-centred approach to dementia and intimate behaviour, rather than the deficit-based biomedical model of sexual consent capacity that has tended to dominate dementia care in RACFs to this point [20]. Of course, communicating with residents about their sexual needs (which was not commonly reported in this study) would be a more direct resident-centred approach.

Staff wanted to intervene in a manner that was respectful. For some, this meant prioritising the needs, rights, dignity, and decision-making capacity of the residents in the relationship, even though one resident had mild cognitive impairment. It also meant being discrete when discussing the relationship. For others, acting in a way that they thought was respectful instead ran the risk of being disrespectful to the residents in the relationship. For instance, many staff mentioned that they would defer to the wishes of family, or other residents, if there were objections to the couple’s relationship or displays of affection. This reflects the tension of private living in a public space [19], but also indicates that at times staff may act in such a way as to ‘keep the peace’ with other residents and family members rather than upholding resident rights and supporting intimacy.

Distracting, or diverting the resident’s attention, is a strategy that is often used as a first-line non-pharmacological treatment for challenging behaviour in people with dementia [3], including sexually inappropriate behaviour [10]. The commonly identified use of distraction by staff in this study suggests that they viewed the resident relationship as ‘inappropriate’ rather than a normal expression of need, as is often the case in RACFs [11, 28]. This further highlights the education deficits of staff and opportunities for improvement.

Similarly, separating, which was also cited as strategy to be implemented, can have the unintended consequence of causing psychological harm [39]. Previous research has found separation to be a common response to sexuality involving a resident with dementia even when there is no suggestion of abuse/harm [38]. Given the psychological health benefits associated with later life relationships, such as enjoyment and improvements in wellbeing and quality of life [31], separation and its use in preventing supportive relationships in aged care could be considered a form of physical restraint. Adopting a protective paradigm is not without consequence.

Previous research has highlighted the absence of formal policies in RACFs regarding resident sexuality, with those that do exist often found to be lacking in detail [23, 25]. The implications of this oversight are considerable,without a policy to guide practice, staff are more likely to approach resident sexuality as ‘non-normative’ and ‘problem behaviour’ [11, 28]. The considerable variability reported in the current study in terms of the way staff respond to an intimate relationship involving residents and cognitive impairment further emphasises the need for urgent policy development in this area in order to better support staff and provide person-centred care that meets the individual needs of residents.

This study was not without limitations. We set out to learn how aged care staff would intervene in a hypothetical relationship, however how these same staff would act in reality remains untested. We were also interested in learning more about the reasons why staff would intervene; respondents largely did not disclose their motivations, making it difficult to interpret whether some actions were driven by a supportive or prohibitive stance (e.g., ‘discuss’ with residents is ambiguous and could imply a discussion of how to support needs or contrastingly a discussion where residents are reprimanded). The study design (i.e., survey) did not allow us to follow up with participants to obtain this information. The inclusion of only senior staff in our sample, who reported a high level of education regarding sex and the older person, also limits the findings. It is possible, and even likely probable, that staff cohorts with less education and/or experience may give different weight to the various reported interventions when faced with a situation akin to the hypothetical situation described in this study. Response bias is also a consideration; it is possible that the staff who accepted the survey invitation were more likely to hold favourable views of resident sexuality than the wider population of senior aged care staff.

Strengths of this study include its broad approach to recruitment, with all RACFs in Australia invited to participate. The study contributes to the small but growing literature on sexuality in Australian residential aged care and highlights the need for policy development in this area. Future research could examine whether the gender of a person living with dementia and entering a sexual relationship impacts staff responses to the situation and intended interventions. Future research could also be directed towards the views of residents’ families regarding sexual relationships in residential aged care, which this study found were influential in the decision-making process for many staff.

## Conclusions

Older people living with dementia in RACFs have the right to sexual expression and to engage in positive intimate relationships. This study highlights the considerable variability in the responses of RACF staff to an intimate relationship between residents when one resident has cognitive impairment. While many staff are supportive and adopt a resident-centred approach, other approaches deny residents and may induce rather than curtail harm. There is a need for policy development in this area in order to better support staff and provide person-centred care that meets the individual needs of residents.

## Data Availability

The data that support this study cannot be publicly shared due to ethical and privacy reasons (consent for this purpose was not provided by participants at the time of the study).
